# Splenomesenteric bypass as revascularisation technique after iatrogenic injury of the superior mesenteric artery during radical nephrectomy: A case report

**DOI:** 10.1016/j.ijscr.2019.05.026

**Published:** 2019-05-30

**Authors:** Catarina Afonso, Jorge Pereira, Pedro Eufrásio, Júlio Constantino, Paulo Rebelo

**Affiliations:** a Serviço de Cirurgia do Centro Hospitalar Tondela-Viseu, Portugal; b Serviço de Urologia do Centro Hospitalar Tondela-Viseu, Portugal

**Keywords:** Superior mesenteric artery injury, Splenomesenteric bypass, Nephrectomy, Renal tumor

## Abstract

**Introduction:**

Iatrogenic vascular injuries during radical nephrectomy are rarely reported. In the case of an injury of the superior mesenteric artery, the consequences for the patient are potentially catastrophic. It occurs more frequently in patients with large renal tumors, due to the presence of bulky perihilar adenopathies, or in cases of pyelonephritis. In most cases, the inadvertent injury of the artery occurs due to the difficulty in distinguishing it from the left renal artery.

**Case:**

69-year-old male, with a malignant neoplasm of the left kidney with the involvement of the tail of the pancreas, tumor thrombus in the left renal vein and multiple left para-aortic adenopathies, whose histological examination revealed to be a renal sarcomatoid carcinoma pT4N1M0G3. A radical nephrectomy was performed with caudal splenopancreatectomy and left paraaortic lymphadenectomy, with an iatrogenic injury of the superior mesenteric artery at its origin. A terminoterminal anastomosis was performed from the proximal stump of the splenic artery. The postoperative period went uneventfully. Control imaging in the follow-up showed permeability of the celiac trunk and the superior mesenteric artery.

**Conclusions:**

Intra-operative superior mesenteric artery injury should be promptly identified and repaired to prevent gut ischemia and all its dire consequences. There are different repair options, and the technique should be chosen according to the degree of injury. The splenomesenteric bypass has numerous advantages.

## Highlights

• Iatrogenic injury of the superior mesenteric artery is rare.
• This injury imparts a high risk of mortality due to visceral ischemia and death.
• An anastomosis to the splenic artery may be an easier solution than an aorto-mesenteric bypass.

## 1 Introduction

Renal cell carcinoma is a common malignant tumor of the urinary system and constitutes 3% of all solid neoplasms. Is a highly vascular neoplasm, preferentially invading the venous system. Invasion of the inferior vena cava and renal veins occurs in 4–10% of patients at diagnosis. Many of these tumors are of low grade and surgical treatment, that is, nephrectomy with thrombectomy offers the best chance of cure and survival. The last edition of the TNM staging system classifies these patients in two distinct categories: T3b when the tumor invades the renal veins or the inferior vena cava or its wall below the diaphragm, or T3c when there is extension of the tumor to the inferior vena cava above the diaphragm [1,2].

Multiple classification systems are used to characterize venous involvement. Mayo’s classification is commonly used and consists of four categories depending on the extent of the thrombus. Level I: the thrombus is limited to the renal vein or <2 cm of the inferior vena cava; level II: the thrombus extends >2 cm above the confluence of the renal vein with the inferior vena cava but inferiorly to the hepatic veins; level III: the thrombus invades the inferior intra-hepatic vena cava and level IV: the thrombus is in the supradiaphragmatic inferior vena cava or in the right atrium [3].

Surgical management should be performed by a multidisciplinary team, including urologists, general or hepatobiliopancreatic surgeons and, if needed, cardiothoracic surgeons. Their role allows improvement of surgical exposure necessary for thrombus and tumor excision and prevention or repair of possible iatrogenic injuries during the procedure [4].

Iatrogenic injury of aortic branches during radical nephrectomy, particularly the superior mesenteric artery, occurs more frequently in patients with large renal tumors requiring extended lymphadenectomy, due to the presence of bulky perihilar adenopathies, or in cases of pyelonephritis [5]. Most reported cases refer to the inadvertent injury or ligation of the superior mesenteric artery during a left nephrectomy because of difficulty in distinguishing it from the left renal artery [5,6]. This is due to the proximity of the ostium of these vessels, corresponding to the danger zone described by Brunnet et al. [7].

Mesenteric artery injury imparts a high risk of mortality due to visceral ischemia. The degree of ischemia depends on the severity and location of the injury. Fullen et al., in 1972, proposed a classification of these injuries based on the anatomical zone affected and the degree of associated intestinal ischemia. Zone 1 is the superior mesenteric artery trunk from the aorta to the first major branch, which is generally the inferior pancreatoduodenal artery, zone 2 comprises the segment between the inferior pancreatoduodenal artery and the middle colic artery, zone 3 refers to the superior mesenteric artery trunk distal to the middle colic artery, and zone 4 involves the segmental branches, jejunal, ileal or colic [8,9]. The more proximal the injury, the more severe is the subsequent intestinal ischemia.

Iatrogenic vascular injuries during radical nephrectomy are rarely reported, possibly being underestimated [6,7,[10], [11], [12], [13]]. In the case of an injury of the superior mesenteric artery, the consequences for the patient are potentially catastrophic. Intra-operative diagnosis and prompt repair are of utmost importance to lower the dismal prognosis [5,7].

The authors report a case of an iatrogenic lesion of the superior mesenteric artery during a radical nephrectomy with distal pancreatectomy and left para-aortic lymphadenectomy.

The present work has been reported in line with the SCARE criteria [14].

## 2 Case report

**Fig. 1:**
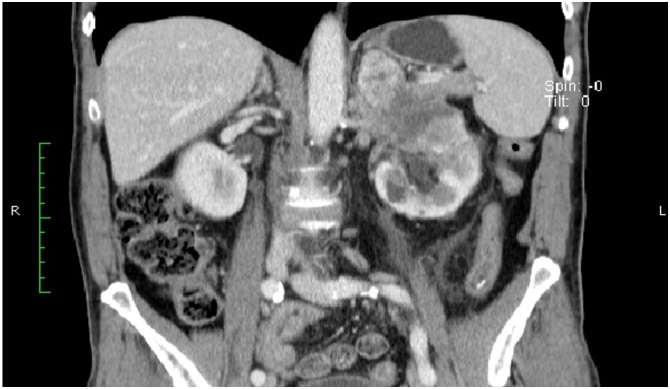
Abdominal CT revealed an 8 cm left renal growth suggestive of neoplasia.

A 69-year-old male patient, with a three months history of abdominal pain asthenia and macroscopic hematuria, was admitted to the outpatient clinic. Abdominal CT revealed an 8 cm left renal growth suggestive of neoplasia, with the involvement of the tail of the pancreas, tumor thrombus in the left renal vein and multiple left para-aortic adenopathies (Fig.1). A biopsy was performed and showed to be inconclusive regarding the possibility of renal cell carcinoma. Radical left nephrectomy with distal pancreatectomy and splenectomy was proposed.

**Fig. 2:**
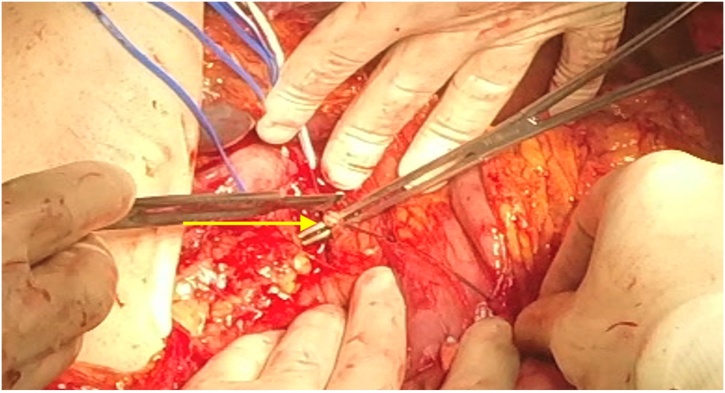
Isolation of the superior mesenteric artery what was thought to be the left renal artery.

**Fig. 3:**
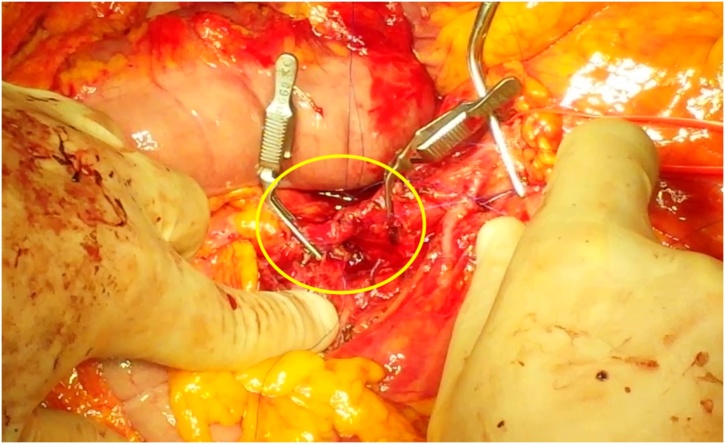
Terminoterminal anastomosis was performed [G1] from the proximal stump of the splenic artery.

The procedure (Video 1 in Supplementary data) started with an extensive Cattel-Braasch Maneuver, allowing exposure of the inferior vena cava and the aorta, and thus isolation of the left renal vessels. The tail of the pancreas and spleen were freed, and a no-touch approach [15] was adopted to minimize the spread of tumor cells with proximal splenic vein ligation. Caudal splenopancreatectomy was performed with section of the pancreas with a GIA 80 (vascular load), followed by proximal ligation and section of the splenic artery and what was thought to be the left renal artery (Fig. 2). The junction of the left renal vein with the vena cava was opened, and the tumor thrombus was resected, followed by caval suture. The renal vessels were, at this point, presumably controlled. The nephrectomy was continued with the release of the kidney and para-aortic lymphadenectomy, during which only the stump of the left renal vein could be identified, lacking the previously ligated renal artery stump. The renal artery was located inside the mass of lymph node tissue in the left para-aortic space, and the stump belonged to the superior mesenteric artery, ligated flush with the aorta. There was no arterial pulse in the mesentery confirming the injury. After removing the specimen, the distal stump of the superior mesenteric artery was exposed, and a repair with a terminoterminal anastomosis was performed from the proximal stump of the splenic artery (Fig. 3). The viability of the gut was assessed by palpation of an arterial pulse in the superior mesenteric artery.

**Fig. 4:**
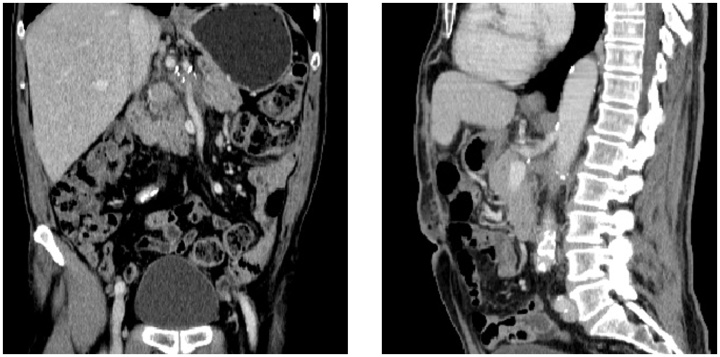
Abdominal CT at three months showed permeability of the celiac trunk and the superior mesenteric artery.

The postoperative period went uneventfully. Histological examination showed a renal sarcomatoid carcinoma pT4N1M0G3. Control imaging at three months showed permeability of the celiac trunk and the superior mesenteric artery (Fig. 4).

## 3 Discussion

During a radical nephrectomy, in the presence of bulky tumor mass, correct identification of the branches of the aorta may be technically challenging, but of extreme importance. However, the size of the mass distorts normal anatomy, and the presence of adenopathies and adhesions makes it challenging to identify anatomical structures [6,8]. Radical nephrectomy requires extensive dissection with need of attention to multiple arterial collaterals usually found in these tumors. It should be borne in mind that the anatomical variations of renal arteries are also relatively frequent [5].

The choice of the best surgical approach depends on the level of venous invasion. In this particular patient, it presented invasion of the left renal vein to its confluence with the inferior vena cava. Although the tumor was of the left kidney, a Cattell-Braasch maneuver helped to access the inferior vena cava and to perform thrombectomy safely. This technique allows excellent access to retroperitoneal structures, as the infra-hepatic inferior vena cava, the abdominal aorta below the superior mesenteric artery, and retroperitoneal organs of the right side [12,13].

Iatrogenic injury of aortic branches during abdominal surgery is rare and probably, under-reported. Only very small series and single cases have been reported [[6], [7], [8]]. Moreover, most of these reports include cases of injury to the superior mesenteric artery or the celiac trunk. Some argue that laparoscopy has added risk of injury during nephrectomy [16,17].

There are different repair options, and the technique should be chosen according to the degree of injury. A simple suture can be performed in superficial injuries. Anterograde or retrograde bypasses with venous or PTFE grafts from the aorta or the common iliac artery, despite technically demanding, are good options, with a 10-year risk of thrombosis up to 20% [8,18,20].

The splenomesenteric bypass has numerous advantages. The need for a single anastomosis allows reduction of operative time with low risk of stenosis. Avoids aortic clamping, thus preventing the risk of cardiac ischemia, posttreatment hypotension, and release of atheromatous plaques. Moreover, these bypasses have excellent long-term patency [8,19].

The possible disadvantage of this technique would be the need for splenectomy. However, the splenomesenteric bypass has been reported several times without splenectomy, relying on collateral circulation through the short gastric vessels [18].

In the case of our patient, the lesion of the superior mesenteric artery corresponds to an injury of zone 1, which made it impossible to perform a direct end-to-end anastomosis. Achieving a single anastomosis to a vessel previously exposed, prevented the need for additional dissection or a more prolonged operative time.

Intra-operative superior mesenteric artery injury should be promptly identified and repaired to prevent gut ischemia and all its dire consequences. Delay in diagnosis and treatment remain the main factors associated with high mortality [20].

## 4 Conclusion

This report presents a case where a superior mesenteric artery injury was repaired with a direct bypass of the splenic artery. Although not always feasible, because of concerns of splenic viability, it is an option with several significant advantages, such as a single anastomosis, possibility of reducing operative time, avoiding aortic clamping and long-term permeability.

## Conflicts of interest

Nothing to declare.

## Funding

No funding.

## Ethical approval

Clinical case exempt from ethical approval in my institution.

## Consent

Consent written informed consent was obtained from a relative of the patient for publication of this case report and accompanying images. A copy of the written consent is available for review by the Editor-in-Chief of this journal on request.

## Author’s contribution

Catarina Afonso – Study design, data collection, writing the paper.

Jorge Pereira – He is a senior general surgeon. He is one of the surgeons who performed the surgery. Data collection obtain images, review manuscript.

Pedro Eufrásio – He is an urologist. He is one of the surgeons who performed the surgery.

Júlio Constantino – He is a general surgeon. He is one of the surgeons who performed the surgery.

Paulo Rebelo – He is a senior urologist. He is one of the surgeons who performed the surgery.

## Registration of research studies

Clinical case report, not formal research project.

## Guarantor

Jorge Pereira.

## Provenance and peer review

Not commissioned, externally peer-reviewed.
